# EndoQuad: a comprehensive genome-wide experimentally validated endogenous G-quadruplex database

**DOI:** 10.1093/nar/gkad966

**Published:** 2023-10-30

**Authors:** Sheng Hu Qian, Meng-Wei Shi, Yu-Li Xiong, Yuan Zhang, Ze-Hao Zhang, Xue-Mei Song, Xin-Yin Deng, Zhen-Xia Chen

**Affiliations:** Hubei Hongshan Laboratory, College of Life Science and Technology, College of Biomedicine and Health, Interdisciplinary Sciences Institute, Huazhong Agricultural University, Wuhan 430070, PR China; Hubei Hongshan Laboratory, College of Life Science and Technology, College of Biomedicine and Health, Interdisciplinary Sciences Institute, Huazhong Agricultural University, Wuhan 430070, PR China; Hubei Hongshan Laboratory, College of Life Science and Technology, College of Biomedicine and Health, Interdisciplinary Sciences Institute, Huazhong Agricultural University, Wuhan 430070, PR China; Hubei Hongshan Laboratory, College of Life Science and Technology, College of Biomedicine and Health, Interdisciplinary Sciences Institute, Huazhong Agricultural University, Wuhan 430070, PR China; Hubei Hongshan Laboratory, College of Life Science and Technology, College of Biomedicine and Health, Interdisciplinary Sciences Institute, Huazhong Agricultural University, Wuhan 430070, PR China; Hubei Hongshan Laboratory, College of Life Science and Technology, College of Biomedicine and Health, Interdisciplinary Sciences Institute, Huazhong Agricultural University, Wuhan 430070, PR China; Hubei Hongshan Laboratory, College of Life Science and Technology, College of Biomedicine and Health, Interdisciplinary Sciences Institute, Huazhong Agricultural University, Wuhan 430070, PR China; Hubei Hongshan Laboratory, College of Life Science and Technology, College of Biomedicine and Health, Interdisciplinary Sciences Institute, Huazhong Agricultural University, Wuhan 430070, PR China; Shenzhen Institute of Nutrition and Health, Huazhong Agricultural University, Shenzhen 518000, China; Shenzhen Branch, Guangdong Laboratory for Lingnan Modern Agriculture, Genome Analysis Laboratory of the Ministry of Agriculture, Agricultural Genomics Institute at Shenzhen, Chinese Academy of Agricultural Sciences, Shenzhen 518000, China

## Abstract

G-quadruplexes (G4s) are non-canonical four-stranded structures and are emerging as novel genetic regulatory elements. However, a comprehensive genomic annotation of endogenous G4s (eG4s) and systematic characterization of their regulatory network are still lacking, posing major challenges for eG4 research. Here, we present EndoQuad (https://EndoQuad.chenzxlab.cn/) to address these pressing issues by integrating high-throughput experimental data. First, based on high-quality genome-wide eG4s mapping datasets (human: 1181; mouse: 24; chicken: 2) generated by G4 ChIP-seq/CUT&Tag, we generate a reference set of genome-wide eG4s. Our multi-omics analyses show that most eG4s are identified in one or a few cell types. The eG4s with higher occurrences across samples are more structurally stable, evolutionarily conserved, enriched in promoter regions, mark highly expressed genes and associate with complex regulatory programs, demonstrating higher confidence level for further experiments. Finally, we integrate millions of functional genomic variants and prioritize eG4s with regulatory functions in disease and cancer contexts. These efforts have culminated in the comprehensive and interactive database of experimentally validated DNA eG4s. As such, EndoQuad enables users to easily access, download and repurpose these data for their own research. EndoQuad will become a one-stop resource for eG4 research and lay the foundation for future functional studies.

## Graphical Abstract

**Graphical Abstract:**
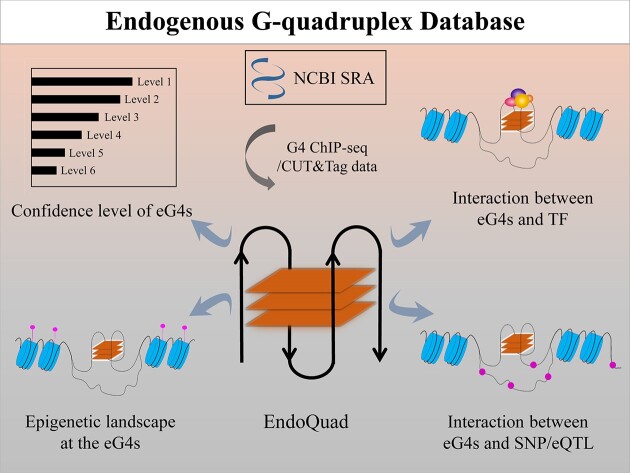

## Introduction

G-quadruplexes (G4s) are four-stranded non-B DNA structures formed in Guanine-rich regions (1–3). G4s are closely associated with gene expression regulation, and both their stimulatory and repressive roles in the genome have been elucidated (2,4–8). Considering the impact of G4 formation or unwinding on gene expression and phenotypes, G4s are regarded as promising therapeutic targets (9–12). Therefore, some small molecules (13–15), oligonucleotides and their analogues (16), and G4 helicases and their binding proteins (17–21) have been developed or identified to control gene expression in therapeutic contexts (22).

With the increasing evidence for the function of G4s, the growing software and algorithms have also been proposed for the genome-wide prediction of potential G-quadruplex-forming sequences (PQSs), which is the first step towards the systematic characterization of G4s (23–30). Based on *in silico* prediction methods, several studies have revealed the evolution and function of G4s (31–34). Inspired by polymerase stop assays, a high-throughput sequencing method named G4-seq has been developed to investigate the abundance and distribution of G4s in the genome (35,36). Further studies have developed G4 chromatin immunoprecipitation sequencing (ChIP-seq) and cleavage under targets and tagmentation (CUT&Tag) technology to map endogenous G4s (eG4s) and to disentangle their regulatory mechanisms (37–42). The term eG4s was used to highlight those G4s that could form quadruplex structures in cells, rather than potential quadruplex forming motifs/sequences (PQSs), and could therefore be detected by specific antibodies. Additionally, some web-based servers have also been established to support eG4 research. For example, there are several databases for the study of G4-associated ligands (G4LDB) (43,44) and interacting proteins (G4IPDB and QUADRatlas) (45,46). G4Atlas contains transcriptome-wide G4s (RNA G4s) by integrating high-throughput sequencing data in human and mouse (47). ONQUADRO and g4db collect only 518 and 28 experimentally determined eG4s, respectively (48,49). Recently, G4Bank was developed, which combines PQSs and a subset of G4 ChIP-seq data and identifies less than 50000 eG4s in the human genome (50). However, they are based on either predicted potential quadruplex-forming sequences (PQSs), only a few eG4s derived from low-throughput methods, or a subset of G4 ChIP-seq data. So far, there is no reference annotation and no comprehensive database of DNA eG4s, significantly limiting the study of eG4 function.

**Figure 1.**
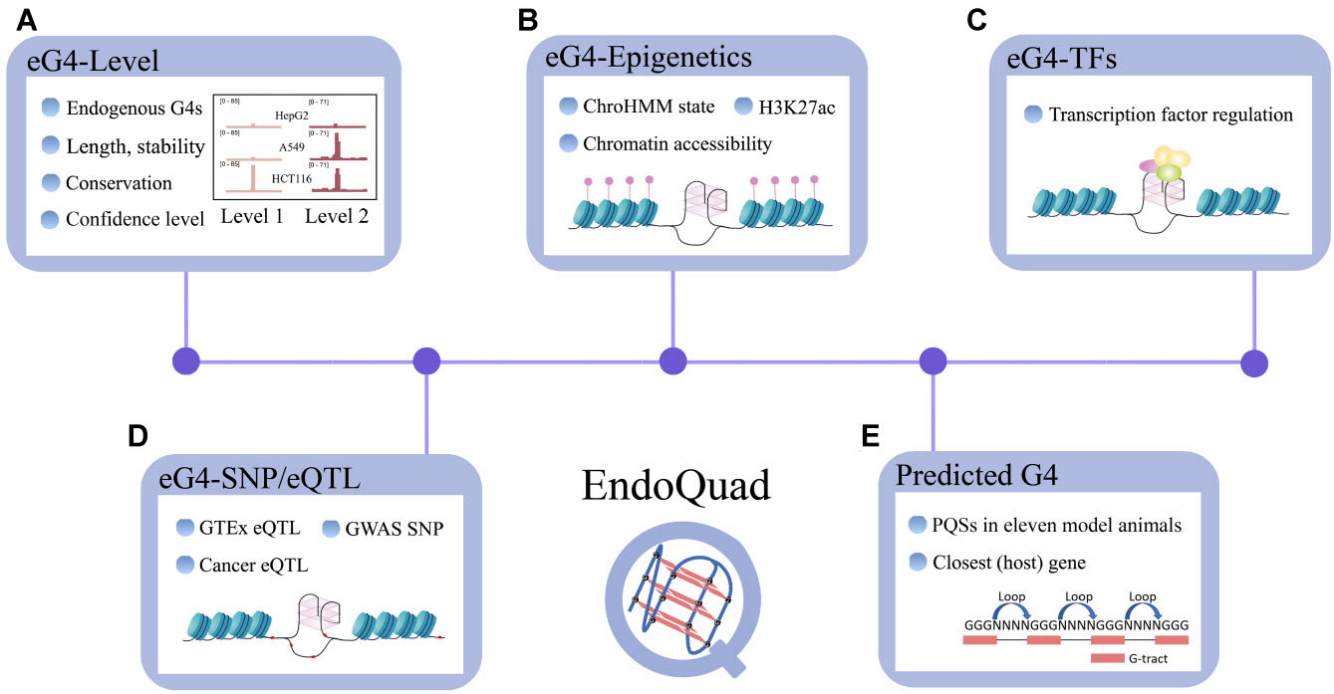
Diagram of the web-based EndoQuad resource. (**A**) Confidence level of eG4s. (**B**) ChromHMM state, histone modification and chromatin accessibility at eG4 loci. (**C**) Regulatory network between transcription factors (TFs) and eG4s. (**D**) Co-localization of eG4s with disease-related elements. (**E**) Predicted quadruplex sequences (PQSs) based on genome sequence.

Here, we establish the most comprehensive database on genome-wide experimentally validated endogenous DNA G-quadruplexes (eG4) database, EndoQuad (Figure 1, https://EndoQuad.chenzxlab.cn/), to tackle the aforementioned challenges (Table S1). EndoQuad contains eG4s of different confidence levels across three vertebrates (human, mouse and chicken) and a quantitative eG4 regulome with comprehensive functional annotations. We have also incorporated GWAS (genome-wide association study) SNPs/eQTL data and prioritized eG4s with regulatory functions in disease and cancer contexts, casting light on the development of therapeutic targets. Furthermore, EndoQuad generated PQS information using pqsfinder (26) across all model animals and implemented a prediction function that allows users to easily predict PQSs in any genome sequence of interest as pqsfinder web (51). The all-in-one search bar, helpful data visualization, detailed information, extensive external links and downloadable resources, render EndoQuad an open-access, user-friendly genome-wide eG4s database. EndoQuad is designed to accommodate future sequencing data and will be continually updated to better serve the eG4s and gene regulation communities.

## Materials and methods

### Collection and analyses of genome-wide eG4 mapping data

We searched ‘Quadruplex’ for G4 ChIP-seq and G4 CUT&Tag data from NCBI GEO DataSets (https://www.ncbi.nlm.nih.gov/gds) (52) and EMBL-EBI (https://www.ebi.ac.uk/) (53) and extracted metadata information for ChIP-seq samples. Totally, we collected 1180 public G4 ChIP-seq samples and 27 G4 CUT&Tag samples from human, mouse and chicken, spanning 45 cell types. Of these, 1181 (97.8%) samples were from 41 human cell lines. The sample size of each cell type ranged from 1 (Hela-S3 and HepG2) to 30 (K562) with a median of 5. Meanwhile, we filtered and manually curated the sample-matched RNA-seq and ATAC-seq (Assay for Transposase-Accessible Chromatin using sequencing) data. We downloaded all raw fastq files and subjected them to quality control using FastQC (v0.11.9, http://www.bioinformatics.babraham.ac.uk/projects/fastqc) and Trim Galore (v0.6.0, https://www.bioinformatics.babraham.ac.uk/projects/trim_galore/). Then, we applied bowtie2 (v2.3.5.1) (54) for read alignment of ChIP-seq and ATAC-seq samples and HISAT2 (v2.1.0) (55) for that of RNA-seq samples. The genome and annotation files of human (hg19) and mouse (mm10) were downloaded from GENCODE (56), and the equivalent files of chicken (Gallus gallus) were downloaded from ENSEMBL (v107) (57). For accurate quantification and analysis, we only retained high-quality reads with the mapping scores (MAPQ) > 20 by SAMtools view (v1.3.1, options: -Sb -q 20) (58). After the filtering, for ChIP-seq and ATAC-seq data, duplicates were marked and removed using Picard MarkDuplicates (v2.25.7) (http://broadinstitute.github.io/picard/), and genomic regions (‘peaks’) with signal enrichment were called for each sample using MACS2 (v2.2.7.1) subcommand callpeak (59). For data with biological replicates, peak regions were considered as positive if they were confirmed in at least two replicates. For RNA-seq data, gene expression levels were calculated using featureCounts (v2.0.3) (60) and normalized to transcripts per kilobase million (TPM).

### Annotation and characterization of eG4s

Since individual eG4s were typically <100 bp, and a peak region could contain eG4s and flanking regions (61), direct analysis of G4s using peaks would unavoidably overestimate the coverage of eG4s. Thus, we predicted potential quadruplex-forming sequences (PQSs) by using pqsfinder (26,51), which could identify non-canonical PQSs bearing bulges or mismatches in G runs and has better performance on G4-seq data and experimentally observed G4s than other tools (28,35,62). We intersected all the PQSs with G4 peaks using bedtools intersect (v2.25.0) (63). The PQS with ≥1 bp overlap with G4 peaks was defined as eG4, otherwise (without overlap) as non-eG4s. The sliding window strategy was carried out using bedtools subcommand makewindows with 1 kb per window. Chromosomal distribution and distance from eG4s and PQSs to Transcription Starting Sites (TSSs) were analyzed by ChIPseeker (64). We calculated the formation frequency of eG4s among different cell lines or under different conditions (such as wild type and knock out). For example, 123 150 (31.5%) eG4s were detected only once among all cell types. Based on the cell type specificity of eG4s, we divided them into six levels, from level 1 to level 6. Promoter regions were defined as the regions 1.5 kb upstream to 1.5 kb downstream of the TSSs of genes. The fraction of expressed genes over all genes within each level was defined as the fraction of expressed genes.

### Roadmap epigenomics data

Epigenomics data of NIH Roadmap Project were obtained from the data portal (http://egg2.wustl.edu/roadmap/) (65). Specifically, all of the 127 consolidated epigenomes with 15 chromHMM states per epigenome were downloaded (http://egg2.wustl.edu/roadmap/data/byFileType/chromhmmSegmentations/ChmmModels/coreMarks/jointModel/final/all.mnemonics.bedFiles.tgz). DHS (DNase I hypersensitivity) narrow peaks with 53 epigenomes were downloaded (http://egg2.wustl.edu/roadmap/data/byFileType/peaks/consolidated/narrowPeak, the file format is [EID]-DNase.macs2.narrowPeak.gz). H3K27ac narrow peaks with 98 epigenomes were downloaded (http://egg2.wustl.edu/roadmap/data/byFileType/peaks/consolidated/narrowPeak/, the file format is [EID]-H3K27ac.narrowPeak.gz). We intersected each genomic feature with chromHMM states, DHS and H3K27ac using bedtools intersect based on the previous work (66). In addition, a genomic region that was ≥1 bp overlapped with a chromHMM state (or DHS/H3K27ac) was defined as annotated by that state (or DHS/H3K27ac). ATAC-seq peaks around PQSs and eG4s from different levels were plotted using deeptools (67).

### Transcription factors and conservation analyses

The TF binding information was downloaded from GTRD, a database including over 5000 ChIP-seq experiment data of human TFs (68). We analyzed the conservation of eG4s using bigWigAverageOverBed from UCSC genome browser (69) based on the previous study (70). We downloaded the conservation files based on the 100-way phastCons (71) (http://hgdownload.soe.ucsc.edu/goldenPath/hg19/phastCons100way/hg19.100way.phastCons.bw) and phyloP (72) files (http://hgdownload.soe.ucsc.edu/goldenPath/hg19/phyloP100way/hg19.100way.phyloP100way.bw) using UCSC genome browser. To identify structurally conserved eG4s between human and mouse, we used the liftOver utility from the UCSC genome browser (http://hgdownload.soe.ucsc.edu/admin/exe/linux.x86_64/liftOver) to project each human eG4 region to the mouse/chicken genome. If the projected mouse/chicken region overlapped with an eG4 then the human eG4 was considered as structurally conserved. We repeated the above analysis to identify structurally conserved eG4s between human and chicken.

Oncogene list was downloaded from ONGene (73) (http://www.ongene.bioinfo-minzhao.org/ongene_human.txt). Tumor suppressor genes were obtained from TSGene 2.0 (74) (https://bioinfo.uth.edu/TSGene/Human_TSGs.txt). GO enrichment and KEGG (Kyoto Encyclopedia of Genes and Genomes) pathway analysis were performed using GSEApy and KEGG (75,76).

### Overlap of eG4s with SNPs and eQTLs

The list of GWAS SNPs was downloaded from the National Human Genome Research Institute (NHGRI) GWAS catalog (77). Unique SNPs were used for further analysis. The data of eQTLs in normal tissues were obtained from GTEx (78), and the data of eQTLs in tumors across 33 cancer types were downloaded from PancanQTL (79). Bedtools subcommand intersect was used to estimate the overlap between eG4s (PQSs) and SNPs or eQTLs. Fold enrichment was calculated as the observed value divided by the simulated value (average values obtained from 10 simulations). Kaplan–Meier (KM) curves were generated using PancanQTL.

### Database implementation

The EndoQuad database was constructed using Flask, a widely used Python-based backend framework (http://flask.pocoo.org/). MongoDB (version 4.0.10) was used as the primary repository for our data. The web interface was designed and executed with Angular2 (version 12.0.2) and enhanced using Bootstrap (version 4.5.0) and Highcharts (version 11.1.0) to ensure a seamless user experience. Content delivery was facilitated by the Apache server. Our website has been extensively tested on a variety of popular web browsers and Google Chrome was recommended.

## Results

### Identification of experimentally validated genome-wide eG4s

We manually curated all public G4 ChIP-seq (N = 1181) and G4 CUT&Tag (*N* = 24) samples, spanning three vertebrates (human, mouse and chicken) and 45 cell types (see Materials and methods). In total, EndoQuad collected the detailed information of 391 503 human eG4s, 71 791 mouse eG4s and 45 044 chicken eG4s, including genomic position, length, structural stability, confidence level and interplay with histone modifications, transcription factors (TFs) as well as regulatory elements (Figure 1, Supplementary Figure S1). To evaluate the reliability of our eG4 data, we investigated the distribution of eG4 number in each cell lines and observed that median number of eG4s detected in each cell line was 12 827, which was comparable to previous works (Supplementary Figure S2). Moreover, a previous study has conducted G4 ChIP-seq on HaCaT cells and unveiled 10 560 high-confidence eG4 peaks (41). Comparing our eG4 data in HaCaT cells (*N* = 18 505) with high-confidence eG4 peaks shows that >98% (18 164 out of 18 505) of eG4s were overlapped eG4 peaks, indicating the reliability of our eG4 data. We also evaluated the reproducibility of eG4s within and between the cell lines. The result shows that reproducibility rate of eG4s in the same tissue or cell type was 6-fold higher than that in different tissues or cell types (Supplementary Figure S3), suggesting the reliability of eG4s within the same cell line.

**Figure 2.**
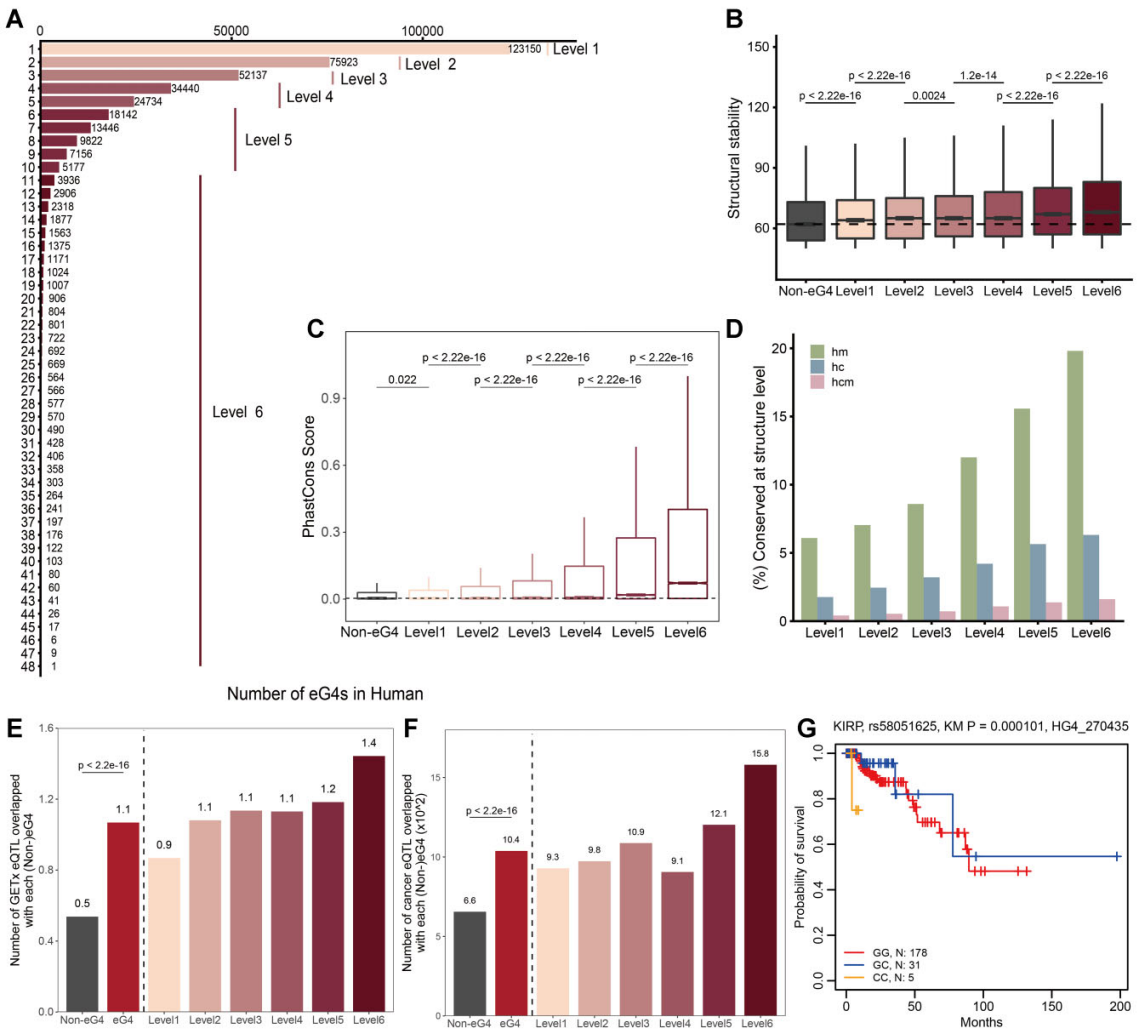
Confidence level and regulation of eG4s. (**A**) Number of eG4s as a function of sample sharing in human using G4 ChIP-seq data. For example, 123150 eG4s are found in only one cell type and 1 eG4 forms quadruplex structures in 48 cell types. Considering the high dynamics of eG4 between different conditions, samples from same cell type but treated with different conditions (e.g. hypoxia or G4 ligand treatment) are regarded as different cell types. The eG4s are classified into 6 levels based on the number of cell types in which they were detected. Comparison of (**B**) structural stability and (**C**) conservation (phastCons score) of eG4s and non-eG4s. (**D**) Proportion of structurally conserved eG4s from each level. h, m and c represent human, mouse and chicken, respectively. For example, hm means the eG4s that are conserved between human and mouse. (**E**) Mean number of eQTLs overlapped with each eG4 or non-eG4. eQTLs in normal tissues were obtained from GTEx. (**F**) Mean number (×10^2^) of cancer eQTLs overlapped with each eG4 or non-eG4. eQTLs in tumors were obtained from PancanQTL. (**G**) Kaplan–Meier (KM) curve was plotted to represent the survival time for rs58051625 in kidney renal papillary cell carcinoma (KIRP). The structural stability was lowered by the eQTL.

Applying the comprehensive reference set of eG4s (Table S2), we explored their occurrence across samples within species. Remarkably, we observed that 123150 (31.5%) human eG4s were detected in only one sample, while mere 16 eG4s were shared by over 45 samples (Figure 2A). Since chicken data contained only one sample, we repeated the analysis in mouse and obtained the similar results, reinforcing the conserved pattern of eG4s (Supplementary Figure S4).

### Confidence levels of eG4s

We assumed that regions detected in multiple samples were more likely to be high-confidence eG4s by mitigating the intrinsic limitations of high-throughput sequencing technologies. Following this principle, we defined confidence levels for the eG4s based on the number of samples in which they were detected. The occurrences in samples from levels 1 to 6 correspond to one, two, three, 4–5, 6–10 and >10 samples, respectively, and the occurrences of human eG4s in different levels are of the same order of magnitude (Supplementary Table S3). To confirm the biological significance of confidence level for future functional studies, we measured the association of confidence level with structural stability, conservation and expression quantitative trait loci (eQTL).

First, we compared the stability scores predicted by pqsfinder, which assigns stability scores based on loop length and guanine number in each track and treated non-eG4s as control. A PQS that overlapped with an eG4 peak in at least one cell type was defined as an eG4 (*N* = 391 503), otherwise (without overlap) as a non-eG4 (*N* = 1 118 756). Our data showed that eG4s in levels 1–6 were more stable than non-eG4s, and the stability of eG4s was gradually increased from level 1 to level 6, and eG4s with higher occurrence were more stable (Wilcoxon test, *P* < 2.2 × 10^−16^) (Figure 2B), confirming that the recurrence of eG4s indicated their validation levels.

Next, we compared their sequence and structure conservation. The sequence conservation was assessed using two metrics, PhastCons (71) and phyloP (72). We found that eG4s were more conserved than non-eG4s, and high-recurrence eG4s were more conserved than low-recurrence ones (Figure 2C, Supplementary Figure S5), suggesting that eG4s, especially recurrent eG4s, might be functional. We then examined eG4 structure conservation, which showed that the eG4s in human cells still formed quadruplex structures in mouse and chicken. In consistent with sequence conservation, recurrent eG4s in human cells were more likely to form eG4s in mouse and chicken (Figure 2D), suggesting their strong structure conservation. Strikingly, more than 33.8% and 5.6% of human eG4s from level 6 exhibited conserved eG4 structures in mouse and chicken, respectively. We also observed that 2362 eG4s were structurally conserved across vertebrates, suggesting that these eG4s might be involved in conserved and core biological processes.

Considering that a comprehensive, multidimensional picture of the contribution of eG4s to epigenetic regulation is still lacking, we integrated chromHMM, a multivariate hidden Markov model trained on histone modifications to identify chromatin states (80), to investigate the contribution of eG4 to epigenetic regulation. Moreover, the combined DHS (DNase I hypersensitivity) and H3K27ac data could define the promoter and enhancer regions, which allows us to investigate the interaction between eG4 and promoter and enhancer. Our data showed that compared with non-eG4s, eG4s showed a higher overlapping proportion with active regulatory chromHMM states (1_TssA, 2_TssAFlnk, 3_TxFlnk, 6_EnhG and 7_Enh), transcribed (4_Tx, 5_TxWk) states and DHS (DNase I hypersensitive sites) and H3K27ac (histone 3 lysine 27 acetylation) peaks (Supplementary Figures S6–S8). Besides, there's a progressive increase in active epigenetic regulation with increasing confidence level (from level 1 to level 6). Furthermore, eG4s were bound by more diverse TFs than non-eG4s, and recurrent eG4s harboured more diverse TFs than nonrecurrent eG4s (Supplementary Figure S9). These observations indicated that recurrent eG4s were involved in complex regulatory programs and might be novel *cis-*regulatory elements in the genome.

To further validate the regulatory potential of eG4s, we extended our scope to eQTLs, a widely used tool to explain the regulatory mechanisms of variants identified by GWAS (81). We intersected eG4s with eQTLs from GTEx and observed that eQTLs exhibited a significant preference for eG4s over non-eG4s, with a more pronounced enrichment of recurrent eG4s than non-recurrent eG4s (Figure 2E). Considering the distinct gene regulation between normal tissues and tumors, we next determined whether the eG4s harbored more cancer-related eQTLs than non-eG4s. Notably, eG4s were still enriched with cancer-related eQTLs (Figure 2F), recapitulating above results (Figure 2E). Since many genes are associated with cancer development and prognoses, these eQTLs overlapped with eG4s might influence the prognosis by altering gene expression. For example, an eQTL named rs58051625 (allele: G/C), located at the promoter of an oncogene *GNAI2*, impaired eG4 structure and exhibited a strong association with the survival time in kidney renal papillary cell carcinoma (KIRP) (Figure 2G).

All these results together demonstrated that the recurrences of eG4s can be used to define confidence levels for functional study, we thus provided the recurrence information as ‘Confidence level’ on the page of ‘eG4-Level’ in EndoQuad.

### EndoQuad development for G4 functional studies

**Figure 3.**
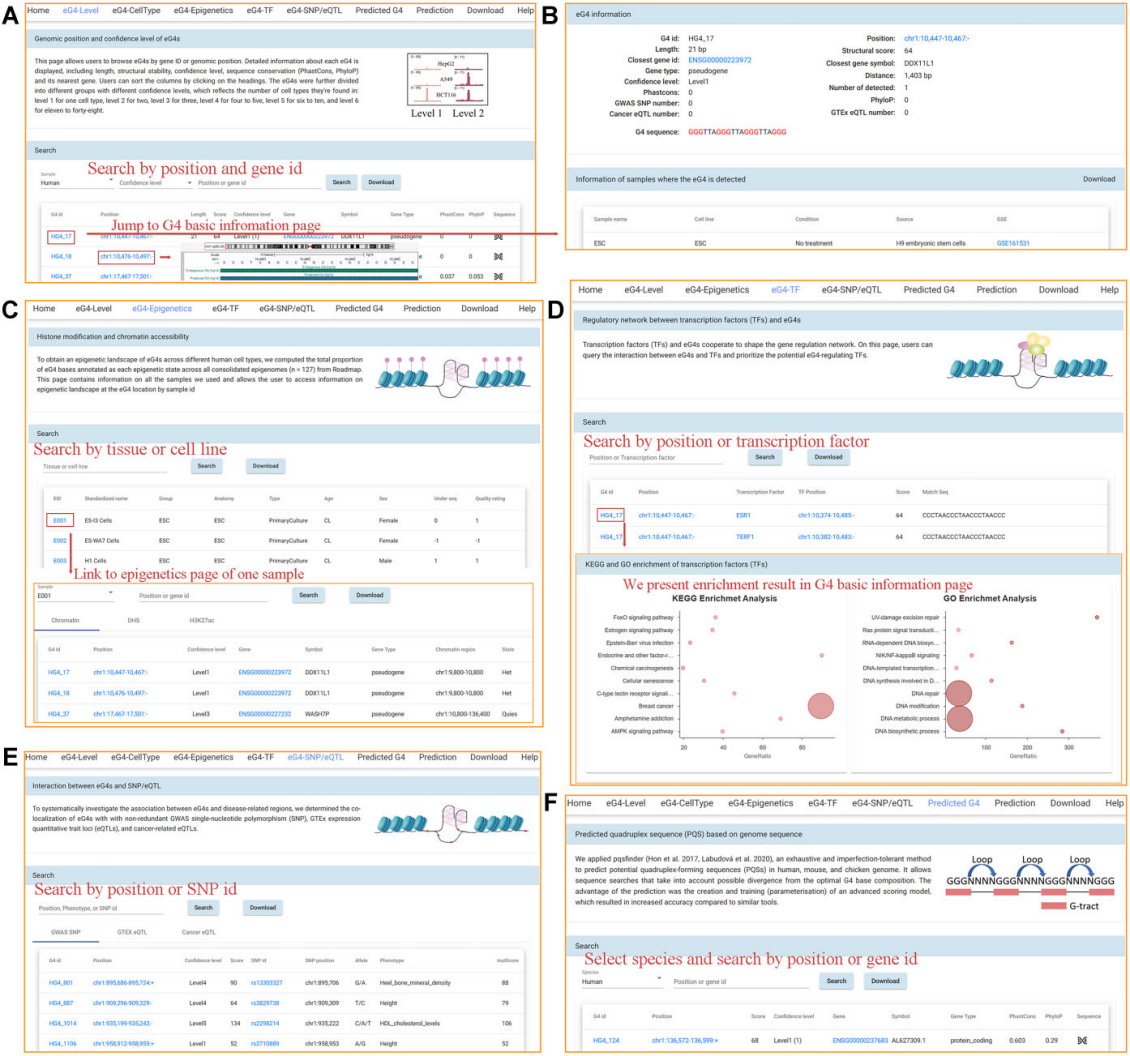
Screenshots of EndoQuad interfaces. (**A**) Browser eG4 confidence level by species, confidence level and genomic position. (**B**) Detailed information includes length, structure score, closest gene, confidence level, evolutionary conservation, and interplay with SNP and eQTL. The results could be visualized in the UCSC genome browser. (**C**) Histone modification at eG4 loci. (**D**) Regulation between transcription factors (TFs) and eG4s. GO enrichment and KEGG pathway of TFs of each G4 are present in dot plot. (**E**) Browser SNP and eQTL at eG4 loci by genomic position or SNP ID. (**F**) Predicted PQSs across model animals.

On this platform, users could query the eG4s by species, confidence level, or genomic position (Figures 1A, 3A–B). The dynamic search results could be visualized in the UCSC genome browser for further analyses (Figure 3A). By entering sample names (as defined by NIH Roadmap Epigenomics Project) or tissues, users could conveniently explore the ChromHMM state, histone modification and chromatin accessibility at eG4 loci (Figure 3C). In addition, EndoQuad allowed users to investigate the interplay between eG4s and TF regulation and provided the GO enrichment and KEGG pathway of TFs of each G4 (Figure 3D). Users could survey the disease/cancer-related eG4s and prioritize eG4s with potential regulatory functions by searching genomic position or SNP ID (Figure 3E). For convenience, we provided the PQSs in all model animals, including human, rhesus, mouse, rat, rabbit, opossum, chicken, zebrafish, fruit fly and worm (Figure 3F). In addition, the prediction function in our database enabled users to easily predict PQSs in any genome sequence of interest. The basic statistical index of the various sessions could be found in Table S4. All the results of an interactive search could be downloaded in a user-friendly manner. Considering the variety of bioinformatic methods for predicting PQSs, we also provided the researchers with the raw G4 ChIP-seq peak files on the download page for researchers to custom identify eG4s if they use other prediction software other than pqsfinder. In summary, the eG4 reference annotation and regulome provided in the resource can serve as a guide for researchers interested in eG4 biology and gene regulation.

## Summary

This study provides a reference annotation of eG4s across vertebrate genomes. In a single framework, we have systematically characterized their intrinsic sequence features, confidence level, genomic distribution, evolutionary conservation, function, regulatory programs and implication in disease biology. As a valuable resource, the user-friendly database EndoQuad will allow researchers to search and repurpose the data in their own eG4 research. Future studies investigating the interaction between eG4s and TF/eQTL will further unravel the regulatory mechanisms of eG4s in the genome.

## Supplementary Material

gkad966_supplemental_files

## Data Availability

All custom computer scripts used in this study are available at https://doi.org/10.6084/m9.figshare.24271450.v1.
