# Supplementary material for: EndoQuad: a comprehensive genome-wide experimentally validated endogenous G-quadruplex database
Source: Nucleic Acids Res. 2023 Oct 30;52(D1):D72–80. doi: 10.1093/nar/gkad966 (PMC10767823; doi:10.1093/nar/gkad966)
Supplement: gkad966_supplemental_files [file gkad966_supplemental_files.zip › Supplementary Figures.docx]

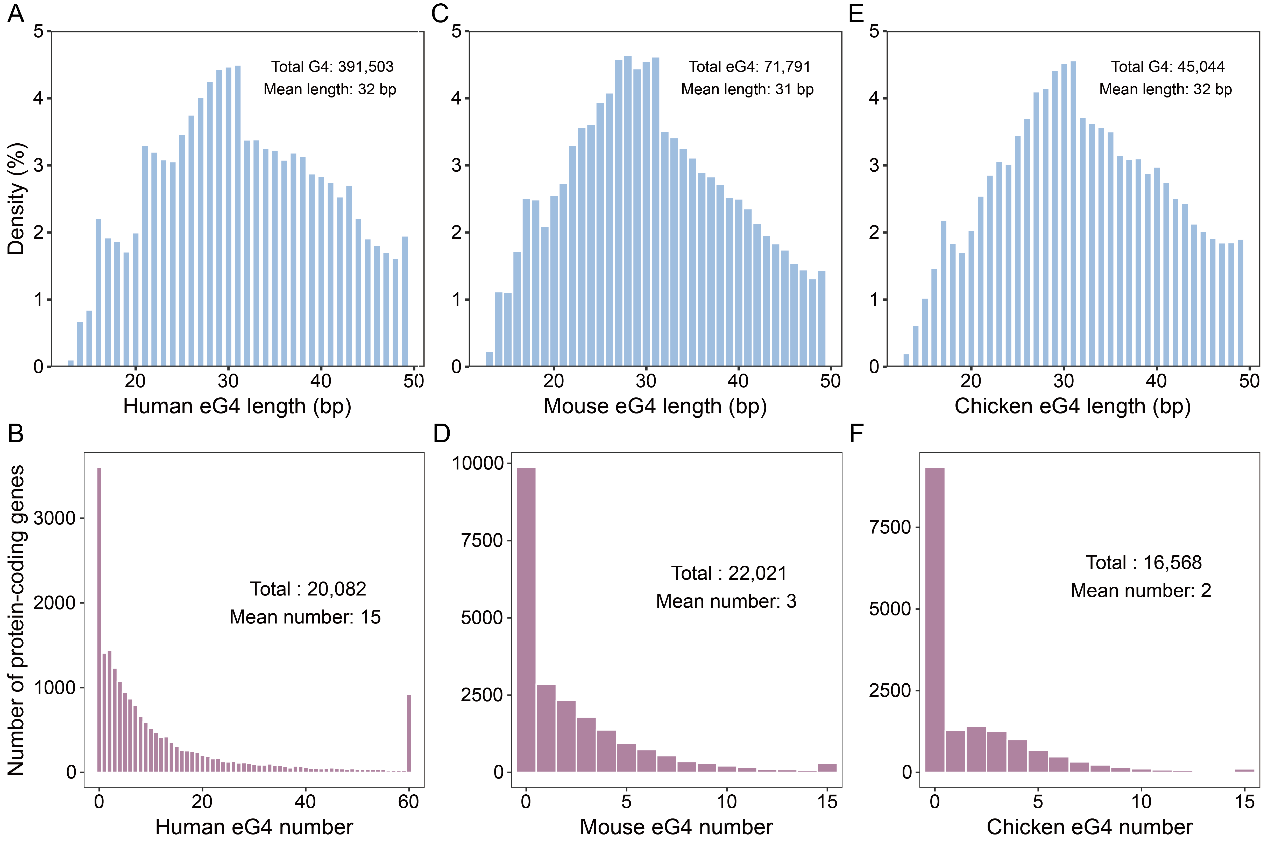


Figure S1. Length distribution of eG4s in human (A), mouse (C), and chicken (E). Distribution of eG4 number contained in each protein-coding gene in human (B), mouse (D), and chicken (F).


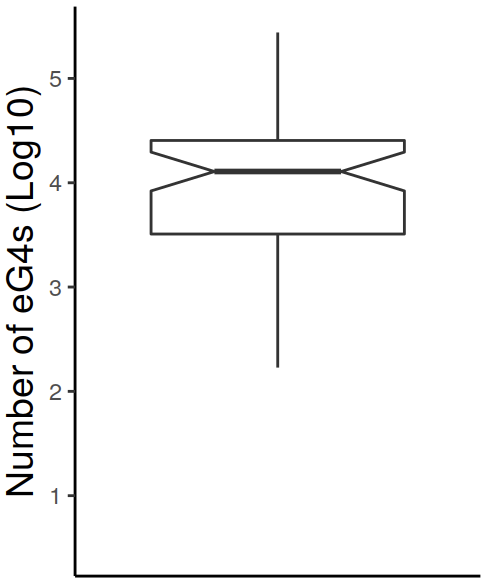


Figure S2. Number of eG4s in each cell lines.


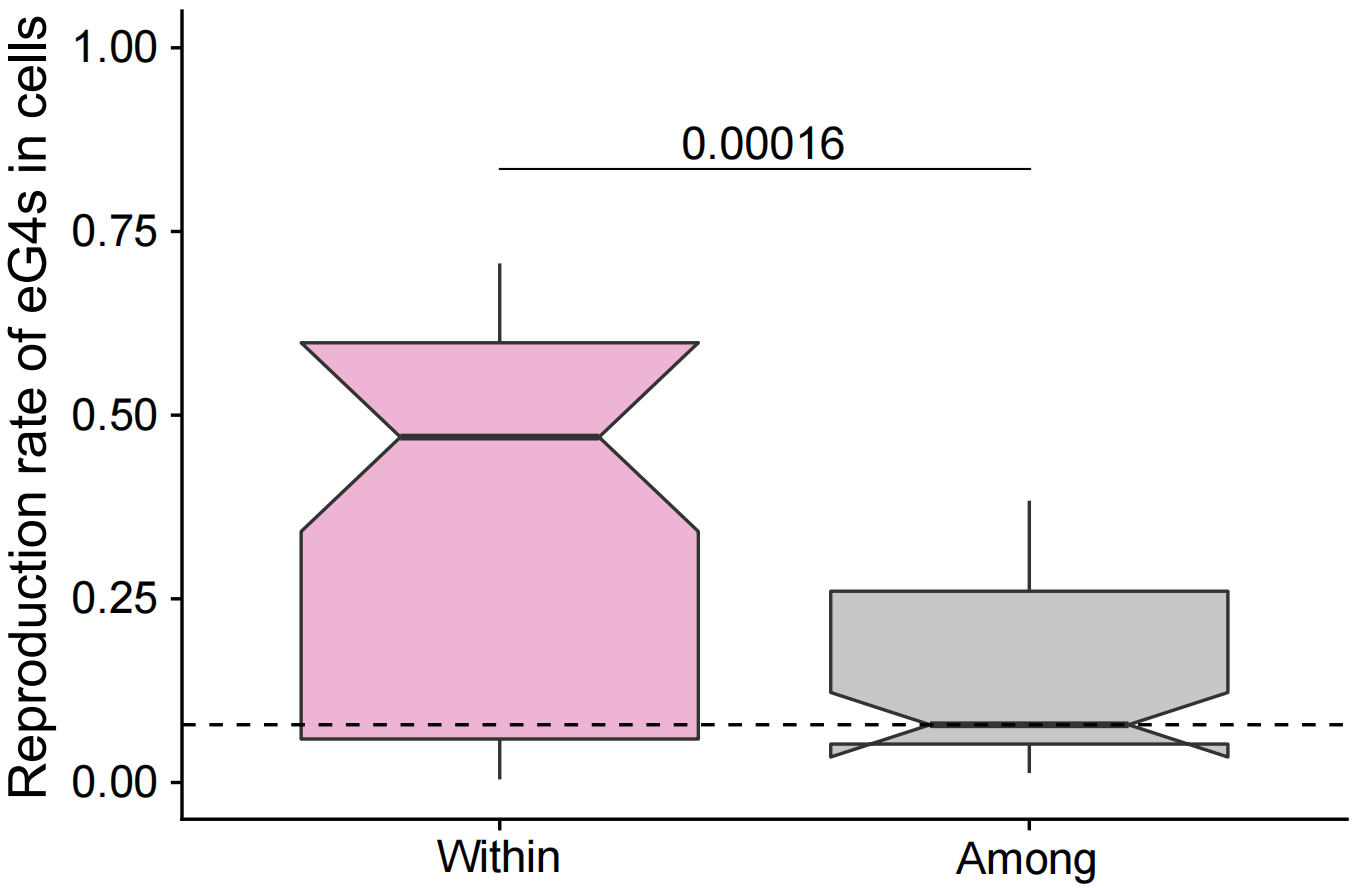


Figure S3. Reproducibility rate of eG4s within and between cell lines.


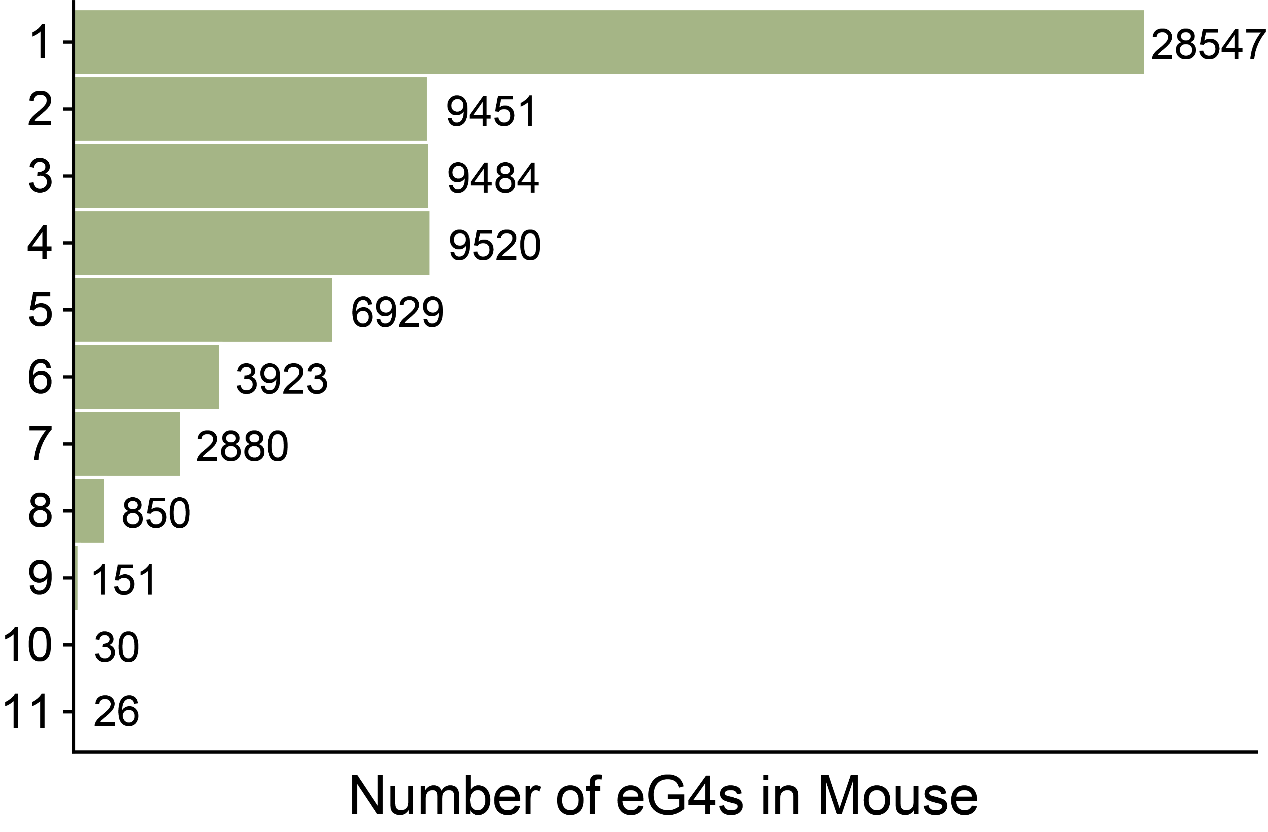


Figure S4. Number of eG4s as a function of tissue sharing in mouse.


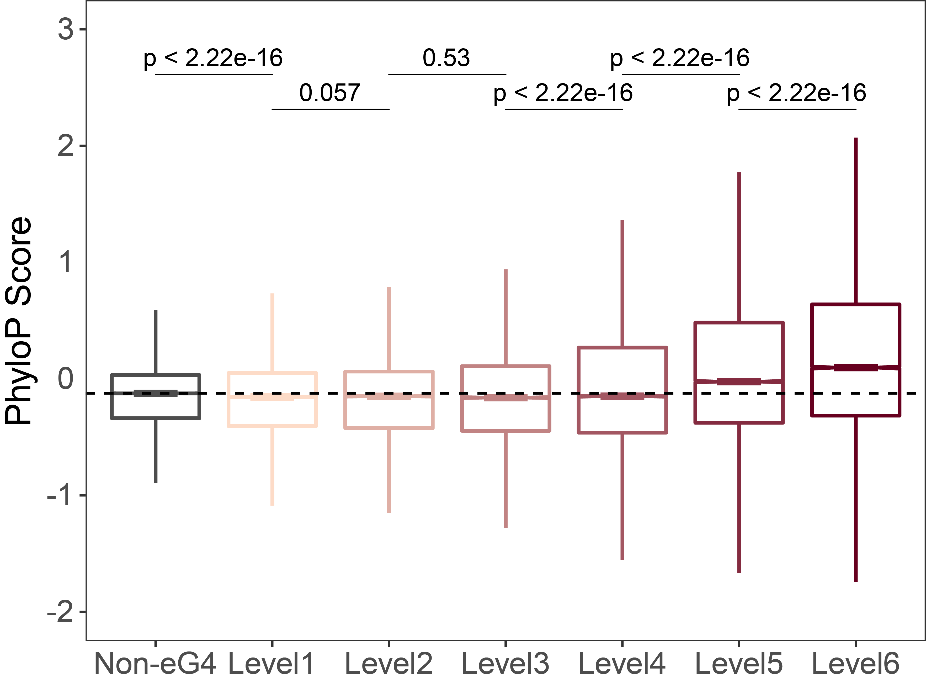


Figure S5. PhyloP score of non-eG4s and eG4s from six levels.


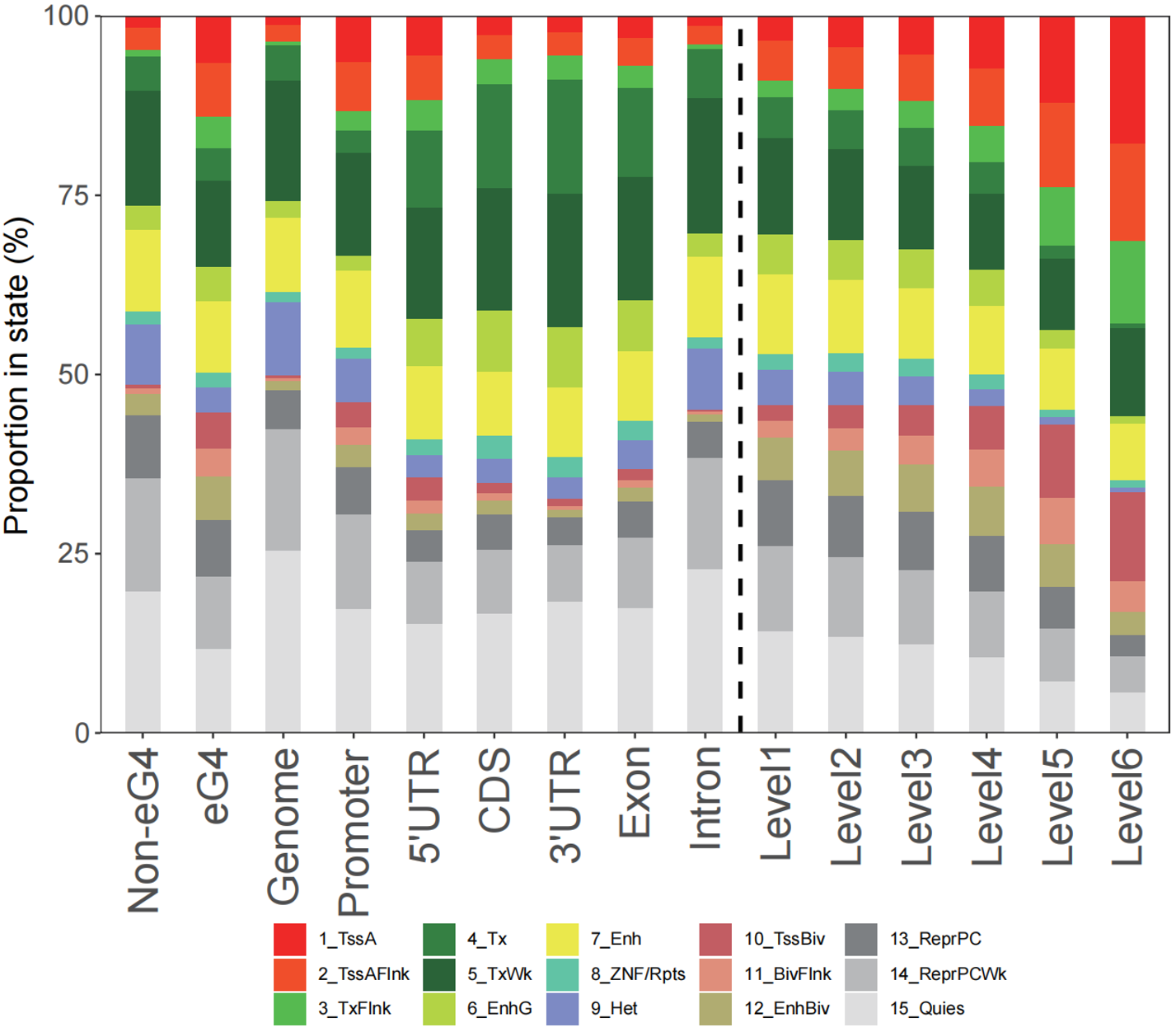


Figure S6. Proportion of bases in eG4s, non-eG4s, and RefSeq genic features annotated with each chromHMM state.


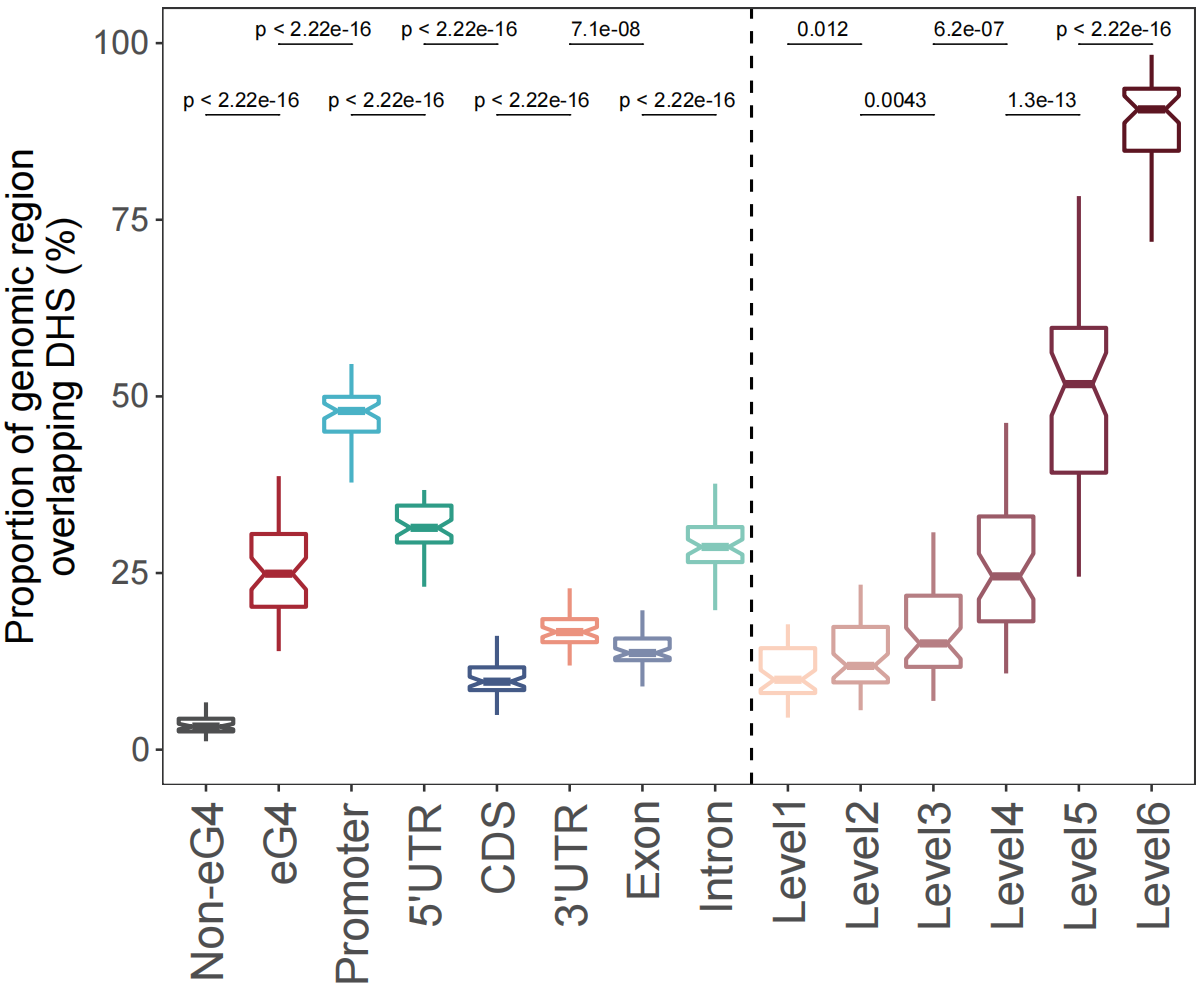


Figure S7. DHS peaks in eG4s, non-eG4s, and RefSeq genic features. All epigenomes were considered (chromHMM states with 127 epigenomes, DHS with 53, and H3K27ac with 98).


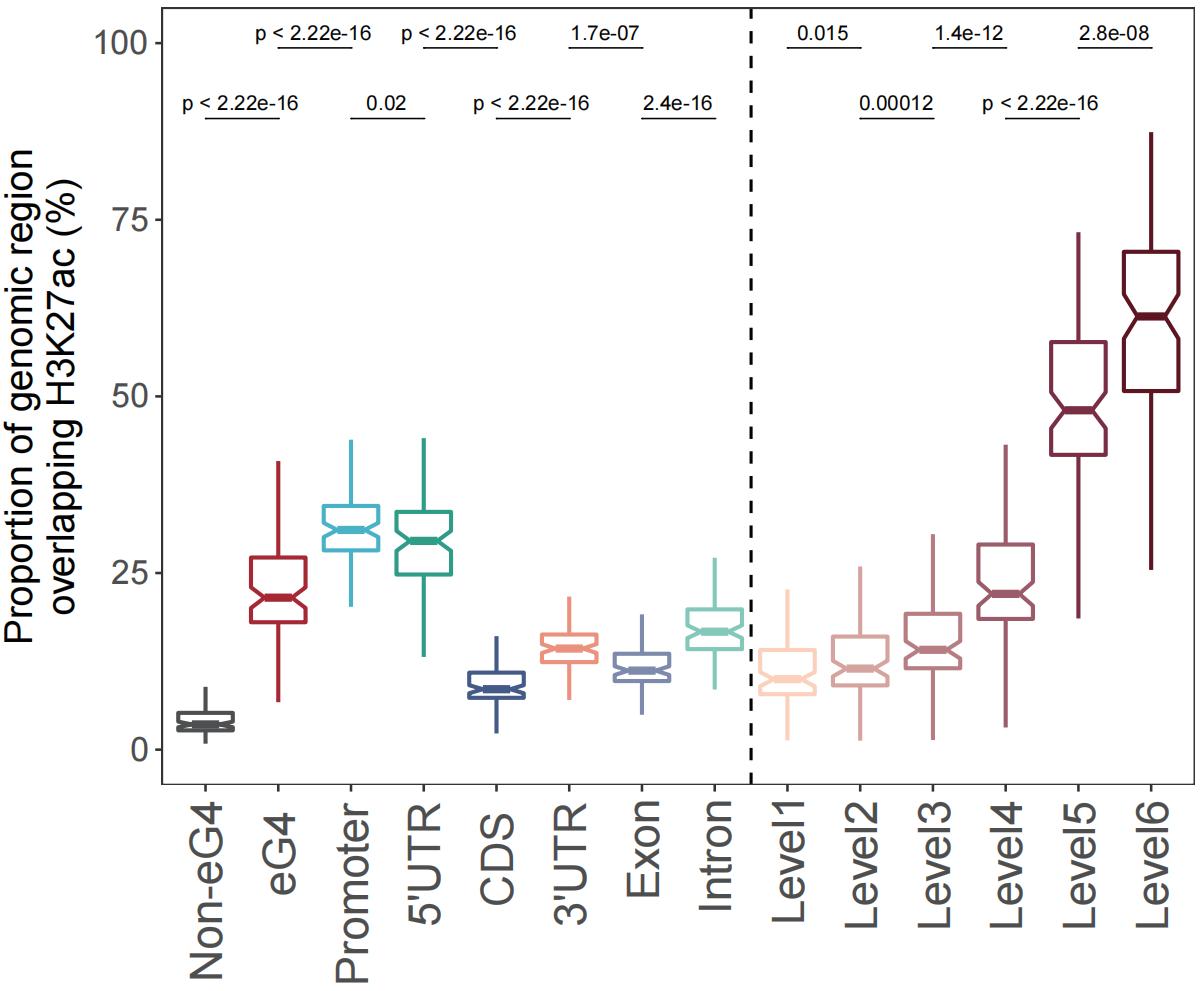


Figure S8. H3K27ac peaks in eG4s, non-eG4s, and RefSeq genic features. All epigenomes were considered (chromHMM states with 127 epigenomes, DHS with 53, and H3K27ac with 98).


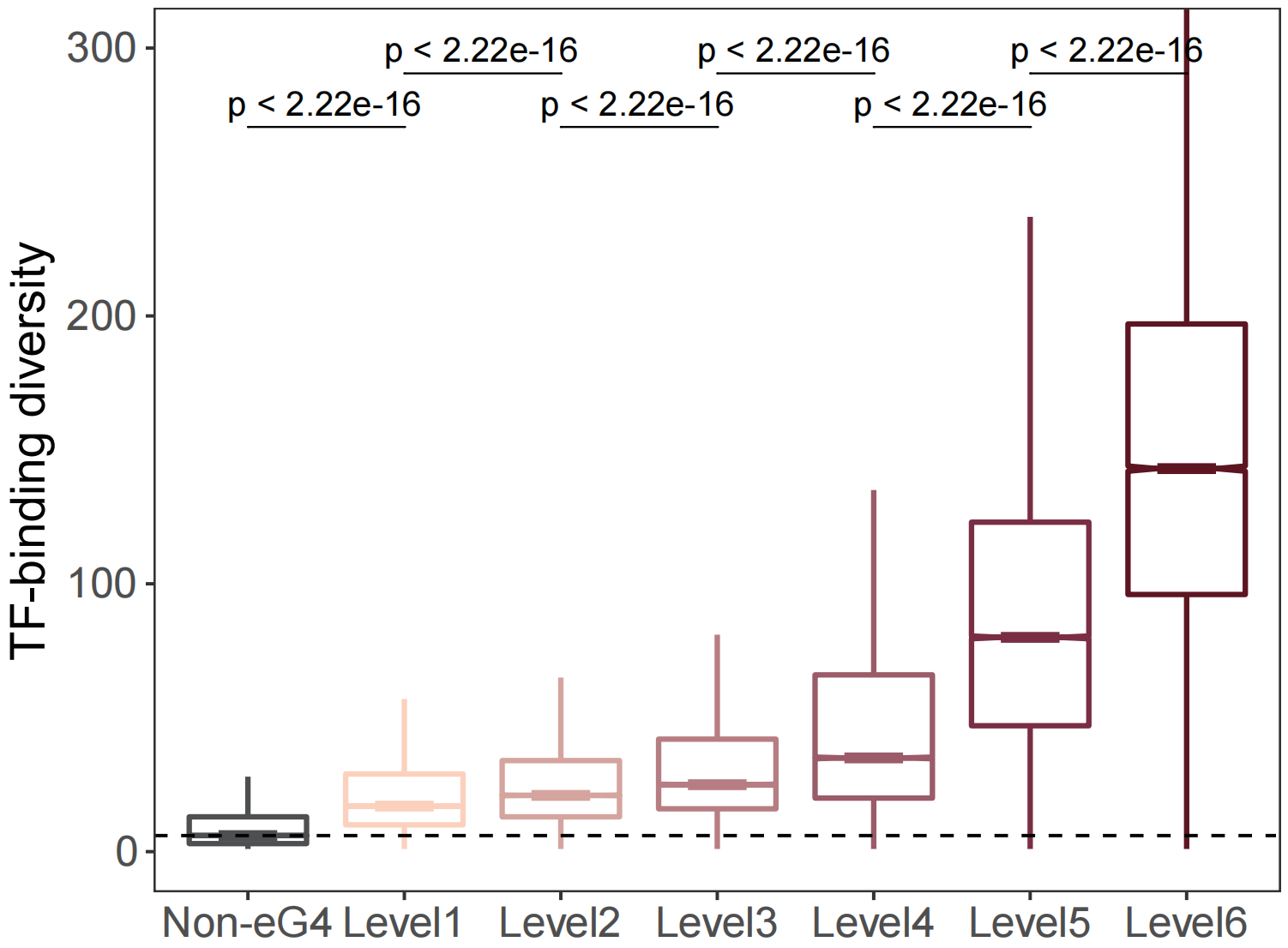


Figure S9. Diversity of TFs overlapping with eG4s. Non-eG4s were set as the control group.
